# Supplementary material for: Study protocol: A cross-sectional survey of clinicians to identify barriers to clinical practice guideline implementation in the assessment and treatment of persistent tic disorders
Source: PLoS One. 2023 Jul 19;18(7):e0288408. doi: 10.1371/journal.pone.0288408 (PMC10355449; doi:10.1371/journal.pone.0288408)
Supplement: S1 File — (PDF) [file pone.0288408.s001.pdf]

Demographics

On behalf of the International Parkinson's Disease and Movement Disorders Society Tic and Tourette Syndrome Study Group, we are requesting you complete this 10 minute survey describing your clinical practices with tic patients.

Practice variations continue to be quite wide and that many barriers in different clinical settings might negatively influence the adoption of the American Academy of Neurology and the European Society for the Study of Tourette Syndrome published guidelines. The objectives of this survey are to 1) identify how clinical practices diverge from the existing AAN and ESSTS guidelines, and 2) to identify categories of barriers leading to these clinical care gaps. There is a need to assess the degree that these clinical guidelines are being followed, contextualize them within diverse practices, and identify barriers encountered so that strategies can be developed to consistently achieve a standard of care.

We sincerely appreciate your time,

Jaclyn M. Martindale, Harini Sarva, Davide Martino, Donald L. Gilbert, Christos Ganos, Tamara Pringsheim, Kevin Black, and Irene A. Malaty on behalf of the Movement Disorder Society Tic and Tourette Study Group

Demographics

Please indicate your specialty	◯ Child Neurology<br>◯ Adult Neurology<br>◯ Child Psychiatry<br>◯ Adult Psychiatry<br>◯ Other
What other specialty?	__________________________________
Are you a movement disorder specialist?	◯ Yes<br>◯ No
Years in specialty practice?	◯ < 5<br>◯ 6-10<br>◯ 11-20<br>◯ 21+ years
Which best describes your practice setting?	◯ Academic clinician<br>◯ Non-academic clinician in a hospital<br>◯ Non-academic clinician in private practice
What is the age range of patients with tic disorders that you currently see in your usual practice?	◯ Exclusively adults<br>◯ Mostly adults<br>◯ Adults and adolescents (12-18 years of age)<br>◯ adolescents, and children (< 12 years of age)<br>◯ Mostly adolescents and children<br>◯ Exclusively adolescents and children
What percentage of patients in your current clinical practice do you see with tic disorders?	◯ 0- 25%<br>◯ 26 - 50%<br>◯ 51-75%<br>◯ 76-100%

In what region do you currently practice?

- ◯ Canada - West Coast [British Columbia]
- ◯ Canada - Northern Territories [Yukon, Northwest Territories, Nunavut]
- ◯ Canada - Prairie Provinces [Alberta, Saskatchewan, Manitoba]
- ◯ Canada - Central [Quebec, Ontario]
- ◯ Canada - Atlantic Provinces [Newfoundland and Labrador, New Brunswick, Prince Edward Island and Nova Scotia]
- ◯ United States - Northeast [Vermont, Connecticut, Delaware, Pennsylvania, Maine, Massachusetts, Maryland, New Hampshire, New Jersey, New York, and Rhode Island]
- ◯ United States - South [West Virginia, Virginia, Texas, Tennessee, South Carolina, Oklahoma, North Carolina, Mississippi, Maryland, Louisiana, Kentucky, Georgia, Florida, the District of Columbia, Delaware, Arkansas, and Alabama]
- ◯ United States - West [Hawaii, Alaska, Idaho, Montana, Colorado, Wyoming, Utah, Nevada, Arizona, California, Washington, Oregon, and New Mexico]
- ◯ United States - Midwest [Minnesota, North Dakota, South Dakota, Iowa, Nebraska, Wisconsin, Missouri, Indiana, Illinois, Michigan, Ohio, and Kansas]
- ◯ Europe - Eastern [Poland, Belarus, Czechia, Slovakia, Hungary, Romania, Moldova, Bulgaria, Ukraine]
- ◯ Europe - Western [Belgium, Luxembourg, Austria, Switzerland, France, Monaco, Liechtenstein, Netherlands, Germany]
- ◯ Europe - Southern [Portugal, Spain, Slovenia, Croatia, Andorra, San Marino, Vatican, Italy, Malta, Bosnia, Herzegovina, Montenegro, Serbia, North Macedonia, Albania, Greece]
- ◯ Europe - Northern [Iceland, Ireland, United Kingdom, Norway, Sweden, Finland, Estonia, Latvia, Lithuania, Denmark]
- ◯ Central America [Belize, Costa Rica, El Salvador, Guatemala, Honduras, Nicaragua, and Panama]
- ◯ Caribbean [the Bahamas, Haiti and the Dominican Republic, Cuba, Jamaica, Puerto Rico, Virgin Islands, Bahamas archipelago, the Windward and Leeward Islands]
- ◯ Mexico
- ◯ South America - Brazil
- ◯ South America - Northern [Colombia, Venezuela, Guyana, Suriname, and French Guiana]
- ◯ South America - Western [Peru, Ecuador, Bolivia]
- ◯ South America - Southern [Argentina, Chile, Uruguay, Paraguay]
- ◯ Africa - Northern [Egypt, Libya, Tunisia, Algeria, Sudan, Western Sahara, and Morocco]
- ◯ Africa - Western [Mauritania, Mali, Niger, Nigeria, Cape Verde, Gambia, Burkina Faso, Benin, Togo, Ghana, Ivory Coast, Liberia, Sierra Leone, Guinea, Guinea-Bissau, and Senegal]
- ◯ Africa - Middle [Chad, Cameroon, eq. Guinea, Sao Tome and Principe, Gabon, Congo, Central African Republic, Democratic Repulbic of the Congo, and Angola]
- ◯ Africa - Eastern [Eritrea, Djibouti, Ethiopia, S. Sudan, Somalia, Kenya, Seychelles, Tanzania, Comoros, Madagascar, Zambia, Mozambique, Mauritius, Uganda, Rwanda, Burundi, Malawi, and Zimbabwe]
- ◯ Africa - Southern [Namibia, Botswana, and South Africa]
- ◯ Asia - Western [Georgia, Armenia, Azerbaijan, Turkey, Cyprus, Syria, Lebanon, Israel, Palestine,

Jordan, Iraq, Oman, Yemen, Kuwait, Bahrain, Qatar, Saudi Arabia]

◯ Asia - Central [Tajikistan, Uzbekistan, Kazakhstan, Turkmenistan, Kyrgyzstan]
◯ Asia - Southern [Sri Lanka, Bangladesh, India, Afghanistan, Pakistan, Bhutan, Nepal, The Maldives, Iran]
◯ Asia - Eastern [China, Mongolia, North Korea, South Korea, Japan, Hong Kong, Taiwan, Macau]
◯ Asia - Southeastern [Brunei, Cambodia, Indonesia, Laos, Malaysia, Myanmar, Philippines, Singapore, Thailand, Timor Leste, Vietnam]
◯ Russia
◯ Australia and New Zealand
◯ Other [free text]

What other region do you practice in?

____

General Treatment

> 75% of the time, if the tics are not functionally impairing to the individual I counsel on watchful waiting:

- ◯ True
- ◯ False

Please specify which is the most common reason why that is not true:

- ◯ I do not feel comfortable with watchful waiting
- ◯ The patient and/or caregiver is not comfortable with watchful waiting
- ◯ I do not commonly see tic patients in my practice without functional impairment
- ◯ I feel treating early leads to better outcomes
- ◯ Other (free text)

Please rate the impact of each of the following on your decision-making when considering treatment initiation for tics in your clinical practice

	Not important	Sometimes important	Mostly important	Always important
If the tics are the most bothersome symptom	◯	◯	◯	◯
Impairment of daily function (school, activities, work)	◯	◯	◯	◯
Type of tics	◯	◯	◯	◯
Social impact of tics	◯	◯	◯	◯
Physical pain or discomfort caused by the tics	◯	◯	◯	◯
Impact on family members	◯	◯	◯	◯

> 75% of the time, I recommend ____ as the initial treatment for tics when needed (check all that apply)

- ☐ Behavioral therapy
- ☐ Medication
- ☐ Psychoeducational support/resources
- ☐ N/a: I refer to another specialty for management
- ☐ N/a: I defer back to the primary care provider/pediatrician for management
- ☐ Other [free text]

What other initial treatment do you recommend?

____

To me, the most important factor in evaluating the ongoing need for tic treatment is:

- ◯ An informal discussion with the patient and/or caregivers
- ◯ Level of functional impairment of tics
- ◯ A change in standardized scales
- ◯ Formal school reports or teacher evaluations
- ◯ Other (free text)

What other factor is important?

____

> 75% of the time, I recommend ____ as the initial treatment for (check all that apply)

	Behavioral therapy	Medication	Psychoeducational support/resources	N/a: I refer to another specialty for management	N/a: I defer back to the primary care provider/pediatrician for management	Other
ADHD	☐	☐	☐	☐	☐	☐
OCD or behaviors	☐	☐	☐	☐	☐	☐
Anxiety	☐	☐	☐	☐	☐	☐
Mood disorders	☐	☐	☐	☐	☐	☐
Disruptive behavior disorder	☐	☐	☐	☐	☐	☐

What other treatment do you initially recommend?

____

Clinical Assessment

When assessing for tic disorders I do the following > 75% of the time (check all that apply)	☐ Evaluate tic severity<br>☐ Evaluate for self-injurious behavior<br>☐ Evaluate for the functional impairment of tics<br>☐ Evaluate for comorbid ADHD<br>☐ Evaluate the impact of ADHD symptoms<br>☐ Evaluate for comorbid OCD<br>☐ Evaluate the impact of OCD symptoms<br>☐ Evaluate for comorbid anxiety<br>☐ Evaluate the impact of anxiety symptoms<br>☐ Evaluate for comorbid mood disorders<br>☐ Evaluate for comorbid disruptive behavior disorders or rage attacks<br>☐ Evaluate for suicidal thoughts or attempts<br>☐ Evaluate the quality of life<br>☐ Evaluate cognitive function<br>☐ Evaluate premonitory urges<br>☐ Other (free text)
What other assessments do you do?	__________________________________

Please rate the barriers to providing all of the above recommendations in routine clinical care

	Never a barrier	Occasionally a barrier	Often a barrier	Always a barrier
Time constraints of clinic	○	○	○	○
Cost of structured interview or standardized scales	○	○	○	○
Accessibility to structured interviews or standardized scales	○	○	○	○
Experience with structured interviews or standardized scales	○	○	○	○
Time commitment of structured interviews or standardized scales	○	○	○	○
Insufficient experience evaluating the comorbid conditions	○	○	○	○
I do not feel comfortable evaluating for the comorbid conditions	○	○	○	○
I do not feel these are all necessary	○	○	○	○
Patients or caregivers fear being diagnosed with these conditions	○	○	○	○

Patients or caregivers fear stigmatization due to these conditions	○	○	○	○

Any other barriers not mentioned which are often or always a barrier to the above recommendations: ______________________________

> 75% of the time, in clinical practice I evaluate tic severity by (check all that apply)

- ☐ Unstructured clinical interview
- ☐ Standardized scales
- ☐ Structured interviews

Which standardized scale do you primarily use in clinical practice:

- ○ Yale Global Tic Severity Scale (YGTSS)
- ○ Modified Rush Video Protocol
- ○ Rush Video-Based Tic Rating Scale
- ○ Parent/Adult Tic Questionnaire (PTQ/ATQ)
- ○ Motor tic, Obsessions and compulsions, vocal tic evaluation survey (MOVES)
- ○ Tourette Disorder Scale (TODS)
- ○ Shapiro Tourette Syndrome Severity (STSS) Scale
- ○ Tourette Syndrome Global Clinical Impression (TS-GCI)
- ○ Other (free text)

What other standardized scale do you use ______________________________

Which structured interview do you primarily use in clinical practice:

- ○ Diagnostic Interview Schedule (DIS)
- ○ Diagnostic Interview Schedule for Children - IV (DISC-IV)
- ○ Schedule for Affective Disorders and Schizophrenia (SADS)
- ○ Kiddie Schedule for Affective Disorders and Schizophrenia (K-SADS)
- ○ Mini-International Neuropsychiatric Interview
- ○ Mini-International Neuropsychiatric Interview for Children and Adolescents (MINI-Kid)
- ○ Structured Clinical Interview for DSM Disorders (SCID)
- ○ Structured Clinical Interview for DSM Disorders, Childhood version (SCID)
- ○ Other (free text)

What other structured interview do you use: ______________________________

To me, functional impairment means (check all that apply):

- ☐ Interfering with daily function at home, school, activities, or work
- ☐ Causing pain or discomfort to the patient
- ☐ Social impact (teasing, bullying, stigmatization)
- ☐ Bothersome to patient
- ☐ Bothersome to caregiver/family

> 75% of the time, in clinical practice I evaluate functional impairment of tics by (check all that apply):

- ☐ Unstructured clinical interview
- ☐ Standardized scales
- ☐ Formal school reports or teacher evaluations
- ☐ Other (free text)

Which standardized scales do you primarily use in clinical practice to evaluate functional impairment of tics:	◯ Yale Global Tic Severity Scale (YGTSS)<br>◯ Tic Accommodation and Reactions Scale (TARS)<br>◯ The Child Tourette's Syndrome Impairment Scale - Parent Report (CTIM - P)<br>◯ Gilles de la Tourette syndrome - QOL Scale (GTS-QOL)<br>◯ Children's - Global Assessment Scale (C-GAS)<br>◯ Other (free text)
Which other standardized scale do you use to evaluate functional impairment of tics	__________________________________
What other way do you evaluate functional impairment of tics in clinical practice?	__________________________________
> 75% of the time, I evaluate for comorbid ADHD by (check all that apply):	☐ Unstructured clinical interview<br>☐ Structured interviews<br>☐ Standardized scales<br>☐ I refer to another specialty for evaluation<br>☐ I defer back to the primary care/pediatrician for evaluation<br>☐ Other (please describe)
Which structured interview do you primarily use in clinical practice to evaluate for ADHD?	◯ Diagnostic Interview Schedule (DIS) or Diagnostic Interview Schedule for Children - IV (DISC-IV)<br>◯ Schedule for Affective Disorders and Schizophrenia (SADS) or Kiddie Schedule for Affective Disorders and Schizophrenia (K-SADS)<br>◯ Mini-International Neuropsychiatric Interview (MINI) or Mini-International Neuropsychiatric Interview for Children and Adolescents (MINI-Kid)<br>◯ Structured Clinical Interview for DSM Disorders (SCID) or Structured Clinical Interview for DSM Disorders Childhood version (Kid-SCID)<br>◯ Other (free text)
Which other structured interview do you use?	__________________________________
Which standardized scales do you primarily use in clinical practice to evaluate for ADHD?	◯ Nolan, and Pelham-IV (SNAP-IV)<br>◯ Conners' Rating Scales<br>◯ ADHD-Rating Scale<br>◯ Wender Utah Rating Scale for ADHD (WURS)<br>◯ NICHQ Vanderbilt Assessment Scales for ADHD<br>◯ Quantified Behavioral Test (QbTest)<br>◯ Other
What other standardized scale do you use to evaluate for ADHD?	__________________________________
What other way do you evaluate for ADHD?	__________________________________

> 75% of the time, in clinical practice I evaluate for comorbid OCD by (check all that apply):

- ☐ Unstructured clinical interview
- ☐ Structured interviews
- ☐ Standardized scales
- ☐ I refer to another specialty for evaluation
- ☐ I defer back to the primary care/pediatrician for evaluation
- ☐ Other (please describe)

What other way do you evaluate for OCD?

____

Which structured interview do you primarily use when evaluating for OCD?

- ◯ Diagnostic Interview Schedule (DIS) or Diagnostic Interview Schedule for Children - IV (DISC-IV)
- ◯ Schedule for Affective Disorders and Schizophrenia (SADS) or Kiddie Schedule for Affective Disorders and Schizophrenia (K-SADS)
- ◯ Mini-International Neuropsychiatric Interview (MINI) or Mini-International Neuropsychiatric Interview for Children and Adolescents (MINI-Kid)
- ◯ Structured Clinical Interview for DSM Disorders (SCID) or Structured Clinical Interview for DSM Disorders Childhood version (Kid-SCID)
- ◯ Other (free text)

What other structured interview do you use when evaluating for OCD?

____

What standardized scale do you primarily use in clinical practice to evaluate for OCD?

- ◯ Children's Yale/ Yale-Brown Obsessive Compulsive Scale (CY/Y-BOCS)
- ◯ Obsessive Compulsive Symptom Inventory (OCI) or Children's Obsessive Compulsive Inventory (CHOCI/CHOCI-R)
- ◯ Florida Obsessive Compulsive Symptom Inventory
- ◯ Leyton Obsessional Inventory Short Form
- ◯ Maudsley Obsessive Compulsive Symptom Inventory
- ◯ Other (please describe)

Which other standardized scale do you use to evaluate for OCD?

____

> 75% of the time, in clinical practice I evaluate for comorbid anxiety by (check all that apply):

- ☐ Unstructured clinical interview
- ☐ Structured interviews
- ☐ Standardized scales
- ☐ I refer to another specialty for evaluation
- ☐ I defer back to the primary care/pediatrician for evaluation
- ☐ Other (please describe)

How else do you evaluate for anxiety?

____

Which structured interview do you primarily use to evaluate for anxiety in clinical practice?	◯ Diagnostic Interview Schedule (DIS) or Diagnostic Interview Schedule for Children - IV (DISC-IV)<br>◯ Schedule for Affective Disorders and Schizophrenia (SADS) or Kiddie Schedule for Affective Disorders and Schizophrenia (K-SADS)<br>◯ Mini-International Neuropsychiatric Interview (MINI) or Mini-International Neuropsychiatric Interview for Children and Adolescents (MINI-Kid)<br>◯ Structured Clinical Interview for DSM Disorders (SCID) or Structured Clinical Interview for DSM Disorders Childhood version (Kid-SCID)<br>◯ Other (free text)
What other structured interview do you use to evaluate for anxiety?	__________________________________
Which standardized scale do you primarily use to evaluate for anxiety in clinical practice?	◯ Generalized Anxiety Disorder (GAD-7)<br>◯ Screen for Adult Anxiety-Related Disorders (SCAARED) or Screen for Child Anxiety-Related Disorders (SCARED)<br>◯ Multidimensional Anxiety Scale for Children (MASC)<br>◯ Beck Anxiety Inventory (BAI) or Beck Youth Inventory (BYI-2)<br>◯ Hamilton Anxiety Scale (HAM-A)<br>◯ Revised Children's Anxiety and Depression Scale (RCADS)<br>◯ Child Behavior Checklist (CBCL)<br>◯ QOL in Neurological Disorders (Neuro-QoLTM) Anxiety or Pediatric Anxiety<br>◯ Other
What other standardized scale do you use to evaluate anxiety?	__________________________________
> 75% of the time, in clinical practice I evaluate for comorbid mood disorders by (check all that apply):	☐ Unstructured clinical interview<br>☐ Structured interviews<br>☐ Standardized scales<br>☐ I refer to another specialty for evaluation<br>☐ I defer back to the primary care/pediatrician for evaluation<br>☐ Other (please describe)
What other way do you evaluate for mood disorders?	__________________________________
Which structured interview do you primarily use to evaluate for mood disorders in clinical practice?	◯ Diagnostic Interview Schedule (DIS) or Diagnostic Interview Schedule for Children - IV (DISC-IV)<br>◯ Schedule for Affective Disorders and Schizophrenia (SADS) or Kiddie Schedule for Affective Disorders and Schizophrenia (K-SADS)<br>◯ Mini-International Neuropsychiatric Interview (MINI) or Mini-International Neuropsychiatric Interview for Children and Adolescents (MINI-Kid)<br>◯ Structured Clinical Interview for DSM Disorders (SCID) or Structured Clinical Interview for DSM Disorders Childhood version (Kid-SCID)<br>◯ Other (free text)

What other structured interview do you use to evaluate for mood disorders?	__________________________________
Which standardized scale do you primarily use to evaluate for mood disorders in clinical practice?	◯ Patient Health Questionnaire (PHQ2 or PHQ9)<br>◯ Beck Depression Inventory (BDI) or Beck Youth Inventory (BYI-2)<br>◯ Children's Depression Inventory (CDI)<br>◯ Quick Inventory Depression Symptoms (QIDS)<br>◯ Hamilton Depression Rating Scale (HDRS)<br>◯ Other
What other standardized scale do you use to evaluate for mood disorders?	__________________________________
> 75% of the time, in clinical practice I evaluate for comorbid disruptive behavior disorders or rage attacks by (check all that apply):	☐ Unstructured clinical interview<br>☐ Structured interviews<br>☐ Standardized scales<br>☐ I refer to another specialty for evaluation<br>☐ I defer back to the primary care/pediatrician for evaluation<br>☐ Other (please describe)
What other way do you evaluate for comorbid disruptive behavior disorders or rage attacks?	__________________________________
Which structured interview do you primarily use to evaluate for comorbid disruptive behavior disorders or rage attacks	◯ Diagnostic Interview Schedule (DIS) or Diagnostic Interview Schedule for Children - IV (DISC-IV)<br>◯ Schedule for Affective Disorders and Schizophrenia (SADS) or Kiddie Schedule for Affective Disorders and Schizophrenia (K-SADS)<br>◯ Mini-International Neuropsychiatric Interview (MINI) or Mini-International Neuropsychiatric Interview for Children and Adolescents (MINI-Kid)<br>◯ Structured Clinical Interview for DSM Disorders (SCID) or Structured Clinical Interview for DSM Disorders Childhood version (Kid-SCID)<br>◯ Other (free text)
What other structured interview do you use to evaluate for comorbid disruptive behavior disorders or rage attacks?	__________________________________
Which standardized scale do you primarily use to evaluate for comorbid disruptive behavior disorders or rage attacks?	◯ Measure of Aggression, Violence, and Rage in Children - Child/Parent Version (MAVRIC-C/P)<br>◯ Rage attack questionnaire- revised<br>◯ Beck Youth Inventory (BYI-2)<br>◯ Cleveland Clinic self-test for Intermittent Explosive Disorder<br>◯ Other
What other standardized scale do you use to evaluate for comorbid disruptive behavior disorders or rage attacks	__________________________________

> 75% of the time, in clinical practice I evaluate for suicidal thoughts and suicide attempts by (check all that apply):	☐ Unstructured clinical interview<br>☐ Structured interviews<br>☐ Standardized scales<br>☐ I refer to another specialty for evaluation<br>☐ I defer back to the primary care/pediatrician for evaluation<br>☐ Other (please describe)
What other way do you evaluate for suicidal thoughts and suicidal attempts?	__________________________________
Which structured interview do you primarily use to evaluate for suicidal thoughts and suicide attempts in clinical practice?	◯ Diagnostic Interview Schedule (DIS) or Diagnostic Interview Schedule for Children - IV (DISC-IV)<br>◯ Schedule for Affective Disorders and Schizophrenia (SADS) or Kiddie Schedule for Affective Disorders and Schizophrenia (K-SADS)<br>◯ Mini-International Neuropsychiatric Interview (MINI) or Mini-International Neuropsychiatric Interview for Children and Adolescents (MINI-Kid)<br>◯ Structured Clinical Interview for DSM Disorders (SCID) or Structured Clinical Interview for DSM Disorders Childhood version (Kid-SCID)<br>◯ Other (free text)
What other structured interview do you use to evaluate suicidal thoughts and suicide attempts?	__________________________________
Which standardized scale do you primarily use to evaluate for suicidal thoughts and suicide attempts?	◯ Patient Health Questionnaire (PHQ9)<br>◯ Beck Scale for Suicide ideation (BSS)<br>◯ Children's Depression Inventory (CDI)<br>◯ Quick Inventory Depression Symptoms (QIDS)<br>◯ Hamilton Depression Rating Scale (HDRS)<br>◯ Columbia Suicide Severity Rating Scale (C-SSRS)<br>◯ Other
What other standardized scale do you use to evaluate for suicidal thoughts and suicide attempts?	__________________________________

Psychoeducation

1) > 75% of the time when initially discussing the diagnosis of tic disorders, I do the following

- ☐ Counsel or provide education on the natural history of tic disorders
- ☐ Counsel or provide education on common comorbid conditions
- ☐ Refer patients and/or caregivers to local support group
- ☐ Refer patients and/or caregivers to online resources such as tourette.org
- ☐ Discuss educational or workplace accommodations

Please rate the barriers to providing all of the above recommendations in routine clinical care:

	Never a barrier	Occasionally a barrier	Often a barrier	Always a barrier
2) I do not feel these are all valuable	○	○	○	○
3) Difficulty in the diagnosis of TS patients	○	○	○	○
4) Time constraints in the clinical setting	○	○	○	○
5) Lack of clinical support staff (social worker, etc) to help coordinate/facilitate	○	○	○	○
6) Availability of local support groups	○	○	○	○
7) Awareness or access to local support groups	○	○	○	○
8) Availability of online resources	○	○	○	○
9) Awareness or access to online resources	○	○	○	○
10) Financial and/or insurance barriers to these resources	○	○	○	○
11) Patients or caregivers fear being diagnosed with these conditions	○	○	○	○
12) Patients or caregivers fear stigmatization due to these conditions	○	○	○	○
13) Lack of school resources to implement supports	○	○	○	○

Behavioral Interventions

> 75% of the time, I prescribe behavioral therapy before or together with medication trials	◯ True<br>◯ False
When prescribing behavioral interventions, I start with CBIT > 75% of the time:	◯ True<br>◯ False
Please specify your top 3 reasons why this is not true	☐ No local CBIT providers<br>☐ Long wait times for local CBIT providers<br>☐ Concern for potential rebound effect of behavioral therapy leading to worsening of tics or onset of new tics<br>☐ Financial cost or insurance coverage of CBIT<br>☐ Patient or caregiver concerns about the time commitment for CBIT<br>☐ Patient or caregiver concerns about travel distance to CBIT<br>☐ Patient or caregiver compliance with CBIT<br>☐ I do not think CBIT is effective<br>☐ I prefer medication<br>☐ I prefer other behavioral therapy over CBIT
What other behavioral therapy do you most often prescribe?	◯ Cognitive behavioral therapy<br>◯ Habit reversal training<br>◯ Relaxation training<br>◯ Exposure and response prevention<br>◯ Dialectical behavioral therapy<br>◯ Online therapy (tichelper.com, online ERP, or online CBIT)<br>◯ Other (free text)
What other therapy was not listed?	__________________________________

When prescribing CBIT, I do the following:

	Never	Sometimes	Most of the time	Always
I discuss with the patient and/or caregivers	◯	◯	◯	◯
I provide a handout on CBIT	◯	◯	◯	◯
I refer the patient and/or caregivers to a resource website (such as tourette.org)	◯	◯	◯	◯
I refer to specific CBIT providers within my institution/practice	◯	◯	◯	◯
I refer to specific CBIT providers outside my institution/practice	◯	◯	◯	◯

I provide the patient and/or caregivers with a list of CBIT providers	◯	◯	◯	◯

If unable to use a local in-person CBIT provider for any reason, I most often do the following

- ◯ I refer to online or video CBIT
- ◯ I provide the patient and/or caregivers with a list of CBIT providers
- ◯ I prescribe cognitive behavioral therapy
- ◯ I prescribe habit-reversal training
- ◯ I prescribe relaxation training
- ◯ I prescribe exposure and response prevention
- ◯ I prescribe dialectical behavioral therapy
- ◯ Other (free text)

What else do you do?

____

Pharmacologic Interventions

> 75% of the time when prescribing medication for tics, I do the following (check all that apply)	☐ Counsel patients or caregivers that treatments infrequently result in complete cessation of tics<br>☐ Confirming failed behavioral therapy prior to initiating medication<br>☐ Counsel on side effects of medication class<br>☐ Assess for comorbid conditions<br>☐ Treat the comorbid conditions if more impairing<br>☐ Start with the lowest dose<br>☐ Prescribe a weight-based dose
My go-to initial treatment for tics is:	☐ Guanfacine<br>☐ Clonidine<br>☐ Topiramate<br>☐ Aripiprazole<br>☐ Haloperidol<br>☐ Pimozide<br>☐ Risperidone<br>☐ Fluphenazine<br>☐ Olanzapine<br>☐ Ziprasidone<br>☐ Quetiapine<br>☐ Tiapride<br>☐ Sulpiride<br>☐ Amisulpride<br>☐ Clozapine<br>☐ Paliperidone<br>☐ Sertindole<br>☐ Iloperidone<br>☐ Lorazepam<br>☐ Midazolam<br>☐ Clonazepam<br>☐ Other (free text)
What other medication is your go-to initial treatment choice?	__________________________________
I prescribe the following class of medications for tics (check all that apply)	☐ Alpha-2-adrenergic agonists<br>☐ Dopamine antagonist or partial agonist (antipsychotics, 1st or 2nd generation)<br>☐ Botulinum toxin injections<br>☐ Topiramate<br>☐ Cannabis-based medication
Which of the following alpha-2-adrenergic agonists do you most commonly use?	☐ Clonidine<br>☐ Guanfacine

> 75% of the time when prescribing alpha-2-adrenergic agonists, I do the following (check all that apply)

- ☐ Counsel AND document on the dual-benefit for individuals with tics and ADHD
- ☐ Counsel AND document potential side effects of alpha-2-adrenergic agonists, such as heart rate, blood pressure or QTc interval
- ☐ Consult with primary care/pediatrician first
- ☐ Consult with a psychiatry or behavioral health specialist first
- ☐ Counsel on the side effects of alpha-2-adrenergic for tics
- ☐ Obtain an EKG before prescribing
- ☐ Obtain EKG periodically during treatment
- ☐ Require clearance by cardiology before prescribing
- ☐ Document that there is no family history of cardiac conditions or QTc abnormalities
- ☐ Document that the patient is not on any other QTc prolonging agents
- ☐ Document that the patient does not have a history of cardiac conditions
- ☐ Taper the medication upon discontinuation to avoid rebound hypertension
- ☐ None of the above

Please rate the barriers to prescribing alpha-2-adrenergic agonists in your practice

	Never a barrier	Occasionally a barrier	Often a barrier	Always a barrier
Patient or parental concerns about side effects	○	○	○	○
Provider concerns about side effects	○	○	○	○
Tolerance of medication/side effects	○	○	○	○
Patient or parental reports of lack of efficacy	○	○	○	○
Financial cost or insurance coverage of medication	○	○	○	○
Access to medication	○	○	○	○
Patient compliance with medication regimen	○	○	○	○
Lack of resources to monitor side effects	○	○	○	○
Lack of experience with this class of medication	○	○	○	○
I do not feel this class of medication is effective	○	○	○	○
I do not think the benefits of this class of medication outweigh the risks	○	○	○	○

Lack of evidence for this medication in tics	○	○	○	○

Which of the following dopamine antagonist or partial agonists (antipsychotics, 1st or 2nd generation) do you most commonly use?

- ☐ Aripiprazole
- ☐ Brexpiprazole
- ☐ Haloperidol
- ☐ Pimozide
- ☐ Risperidone
- ☐ Fluphenazine
- ☐ Olanzapine
- ☐ Ziprasidone
- ☐ Quetiapine
- ☐ Tiapride
- ☐ Sulpiride
- ☐ Amisulpride
- ☐ Clozapine
- ☐ Paliperidone
- ☐ Sertindole
- ☐ Iloperidone
- ☐ Other

Which other dopamine antagonist or partial agonist do you most commonly prescribe?

____

> 75% of the time when prescribing dopamine antagonist or partial agonist (antipsychotics, 1st or 2nd generation) medications, I do the following (check all that apply)

- ☐ Counsel AND document potential side effects such as extrapyramidal, hormonal, cardiac, and metabolic adverse effects
- ☐ Consult with primary care/pediatrician first
- ☐ Consult with a psychiatry or behavioral health specialist first
- ☐ Monitor extrapyramidal symptoms with standardized scales such as the extrapyramidal symptoms rating scale (ESRS)
- ☐ Taper upon discontinuation to avoid withdrawal dyskinesias
- ☐ Require baseline blood work before prescribing
- ☐ Require blood work periodically during treatment
- ☐ Require an EKG before prescribing
- ☐ Require an EKG periodically during treatment
- ☐ Require clearance by cardiology before prescribing
- ☐ None of the above

Please rate the barriers to prescribing dopamine antagonist or partial agonist (antipsychotics, 1st or 2nd generation) agents in your practice

	Never a barrier	Occasionally a barrier	Often a barrier	Always a barrier
Patient or parental concerns about side effects	○	○	○	○
Provider concerns about side effects	○	○	○	○
Tolerance of medication/side effects	○	○	○	○

Patient or parental reports of lack of efficacy	○	○	○	○
Financial cost or insurance coverage of medication	○	○	○	○
Access to medication	○	○	○	○
Patient compliance with medication regimen	○	○	○	○
Lack of resources to monitor side effects	○	○	○	○
I do not have experience with this class of medication	○	○	○	○
I do not feel this class of medication is effective for tics	○	○	○	○
I do not think the benefits of this class of medication outweigh the risks	○	○	○	○
Lack of evidence for its use in tics	○	○	○	○

When prescribing botulinum toxin injection, I do the following:

	Never	Sometimes	Most of the time	Always
I do the injections	○	○	○	○
I refer to a specific provider within my institution/practice	○	○	○	○
I refer to a specific provider outside my institution/practice	○	○	○	○
I provide the patient and/or caregivers with a list of providers that offer botulinum toxin injections for tics	○	○	○	○

What other reason do you recommend botulinum toxin injections for tics?

____

> 75% of the time I counsel on the side effects of botulinum toxin injections, such as temporary weakness and hypophonia

○ True
○ False

> 75% of the time when I recommend botulinum toxin injections for tics when (check all that apply)

☐ Tics are bothersome simple motor tics but are localized/focal
☐ Tics are severely disabling or aggressive vocal tics
☐ If they have failed other adequate medication trials
☐ If they have failed other adequate behavioral therapy trials
☐ Other

Please rate the barriers to prescribing botulinum toxin injections in your practice

	Never a barrier	Occasionally a barrier	Often a barrier	Always a barrier
Patient or parental concerns about side effects	○	○	○	○
Provider concerns about side effects	○	○	○	○
Tolerance of botulinum toxin injections/side effects	○	○	○	○
Patient or parental reports of lack of efficacy	○	○	○	○
Financial cost or insurance coverage of medication	○	○	○	○
Patient or caregiver concerned about the time commitment for botulinum toxin injections	○	○	○	○
Patient or caregiver concerned about the travel distance to botulinum toxin injections	○	○	○	○
Patient compliance	○	○	○	○
Lack of resources to monitor side effects	○	○	○	○
I do not feel botulinum toxin injections are effective for tics	○	○	○	○
I do not think the benefits of this class of medication outweigh the risks	○	○	○	○
I do not follow these patients long term	○	○	○	○
I am not trained to provide this treatment	○	○	○	○
I do not have access to any providers that offer this treatment for tics	○	○	○	○
I was not aware of this treatment for tics	○	○	○	○
Lack of experience with this treatment	○	○	○	○
Lack of evidence for its use in tics	○	○	○	○

> 75% of the time when prescribing topiramate for tics, I do the following (check all that apply)

- ☐ Counsel AND document potential side effects such as cognitive/language, somnolence, weight loss, and increased risk of renal stones
- ☐ Counsel AND document potential interactions with oral contraceptives
- ☐ Consult with the primary care/pediatrician first
- ☐ Consult with a psychiatry or behavioral health specialist first
- ☐ Require blood work before prescribing
- ☐ Require blood work periodically during treatment
- ☐ Require weight checks at home or the primary care periodically during treatment
- ☐ Require renal ultrasound before prescribing
- ☐ Require renal ultrasound periodically during treatment
- ☐ None of the above

Please rate the barriers to prescribing topiramate in your practice

	Never a barrier	Occasionally a barrier	Often a barrier	Always a barrier
Patient or parental concerns about side effects	○	○	○	○
Provider concerns about side effects	○	○	○	○
Tolerance of medication/side effects	○	○	○	○
Patient or parental reports of lack of efficacy	○	○	○	○
Financial cost or insurance coverage of medication	○	○	○	○
Access to medication	○	○	○	○
Patient compliance with medication	○	○	○	○
Lack of resources to monitor side effects	○	○	○	○
I do not have experience with this class of medication	○	○	○	○
I do not feel this class of medication is effective for tics	○	○	○	○
I do not think the benefits of this class of medication outweigh the risks	○	○	○	○
Lack of evidence for use in tics	○	○	○	○

> 75% of the time if a patient is self-medicating with cannabis-based medication, I (check all that apply)	☐ Do nothing - I do not feel it is my responsibility if they are self-medicating<br>☐ I educate AND document evidence for or against its use in tic disorders<br>☐ I educate AND document legal issues but do not monitor<br>☐ I educate AND document ethical issues but do not monitor<br>☐ Other
What else do you do if a patient is self-medicating with cannabis-based medication?	______________________________
Does your regional legislation allow you to prescribe cannabis-based medication?	◯ Yes and my institution/employer allows me to<br>◯ Yes but my institution/employer does not allow me to<br>◯ No<br>◯ I'm not sure
> 75% of the time when prescribing cannabis-based medication for treatment-resistant TS, I do the following (check all that apply)	☐ Consult with the primary care/pediatrician first<br>☐ Consult with a psychiatry or behavioral health specialist first<br>☐ Consider if they are self-medicating with cannabis already<br>☐ Counsel AND document on impaired driving ability<br>☐ I educate AND document evidence for or against its use in tic disorders<br>☐ I educate AND document legal issues<br>☐ I educate AND document ethical issues<br>☐ Other (free text)<br>☐ None of the above

Please rate the barriers to prescribing cannabis-based medication in your practice:

	Never a barrier	Occasionally a barrier	Often a barrier	Always a barrier
I only see pediatric patients	◯	◯	◯	◯
Patient or caregiver concerns about side effects	◯	◯	◯	◯
Provider concerns about side effects	◯	◯	◯	◯
Tolerance of medication/side effects	◯	◯	◯	◯
Patient or caregiver reports of lack of efficacy	◯	◯	◯	◯
Financial cost or insurance coverage of medication	◯	◯	◯	◯
I am concerned about legal implications/issues	◯	◯	◯	◯

I am concerned about ethical implications/issues	○	○	○	○
Lack of evidence to use it in tic disorders	○	○	○	○
I do not believe the benefits outweigh the risks	○	○	○	○
I prefer surgical evaluation for treatment-resistant TS	○	○	○	○
I do not have experience with this class of medication	○	○	○	○
I don't believe in it for personal reasons	○	○	○	○
I don't believe it is effective	○	○	○	○

Deep Brain Stimulation

Do you ever recommend DBS evaluation for treatment-refractory TS?	◯ Yes<br>◯ No
> 75% of the time when recommending a DBS evaluation for treatment-refractory TS, I do the following	☐ Verify AND document the patient has failed or has contraindications to multiple classes of medication<br>☐ Verify AND document medication trials were of adequate dose and duration<br>☐ Verify AND document the patient has failed or has contraindications to behavioral therapy<br>☐ Verify AND document behavioral therapy was of sufficient duration<br>☐ Verify AND document secondary etiologies of tics have been ruled out<br>☐ Verify AND document functional tics have been ruled out<br>☐ Expedite the evaluation when the patient has self-injurious tics<br>☐ Considering DBS earlier when a patient has self-injurious tis<br>☐ Require a mental health professional to evaluate the patient<br>☐ Require a formal neuropsychological evaluation<br>☐ Require a multidisciplinary discussion (psychiatrist or neurologist, a neurosurgeon, and a neuropsychologist) for DBS evaluation<br>☐ Other
What else do you do when recommending DBS evaluation?	__________________________

Please rate the barriers to recommending a DBS evaluation for treatment-refractory TS in your practice

	Never a barrier	Occasionally a barrier	Often a barrier	Always a barrier
I only see pediatric patients	◯	◯	◯	◯
I do not have the resources or access to a multidisciplinary team	◯	◯	◯	◯
Lack of experience with DBS evaluation	◯	◯	◯	◯
Lack of knowledge of DBS evaluation	◯	◯	◯	◯
Lack of documentation or prior records of medication/behavioral trials	◯	◯	◯	◯

Time constraints in clinic to adequately review all previous medication and behavioral trials	○	○	○	○
I do not think there is sufficient evidence to use it in tic disorders	○	○	○	○
I do not think the benefits of DBS outweigh the risks	○	○	○	○
I do not feel DBS is effective	○	○	○	○
Lack of access to providers who offer this evaluation	○	○	○	○
Poorly controlled co-occurring conditions	○	○	○	○
Financial cost or insurance coverage of DBS evaluation or surgery	○	○	○	○
Patient or caregiver concerned about the time commitment for DBS evaluation or surgery	○	○	○	○
Patient or caregiver concerned about the travel distance for DBS evaluation or surgery	○	○	○	○

Any additional barriers you face when providing care for patients with tics you would like to mention?

____
