# Supplementary material for: Study protocol: A cross-sectional survey of clinicians to identify barriers to clinical practice guideline implementation in the assessment and treatment of persistent tic disorders
Source: PLoS One. 2023 Jul 19;18(7):e0288408. doi: 10.1371/journal.pone.0288408 (PMC10355449; doi:10.1371/journal.pone.0288408)
Supplement: Attachment — Submitted filename: Response to Reviewers.docx [file pone.0288408.s002.docx]

Dear Reviewers,

Thank you for your suggestions for revisions. We have incorporated all of the revisions into the manuscript.

1. Please ensure that your manuscript meets PLOS ONE's style requirements, including those for file naming.
   a. The style has been adjusted according the templates provided.
   b. Figure nomenclature has been adjusted
2. Thank you for stating the following in the Competing Interests section
   a. Confirmed it does not altered adherence to PLOS One policies
   b. Added recommended statement on cover letter
   c. Updated competing interests’ statement on cover letter
3. One of the noted authors is a group or consortium “Movement Disorder Society Tic and Tourette Study Group”. In addition to naming the author group, please list the individual authors and affiliations within this group in the acknowledgments section of your manuscript. Please also indicate clearly a lead author for this group along with a contact email address.
   a. All of the authors are submitting on behalf of the study group. The first and corresponding author is the lead author for this group. Added the word all in author list to provide clarification.
   b. Added affiliations to author list in acknowledgments
4. Your ethics statement should only appear in the Methods section of your manuscript. If your ethics statement is written in any section besides the Methods, please delete it from any other section.
   a. Moved to methods 154-157
   b. Also removed from abstract
5. Please review your reference list to ensure that it is complete and correct. If you have cited papers that have been retracted, please include the rationale for doing so in the manuscript text, or remove these references and replace them with relevant current references. Any changes to the reference list should be mentioned in the rebuttal letter that accompanies your revised manuscript. If you need to cite a retracted article, indicate the article’s retracted status in the References list and also include a citation and full reference for the retraction notice.
   a. Reviewed references
6. It might be useful to discuss the modified Delhi process briefly in line 105
   a. Expanded upon this in lines 105-108
7. It might also be useful to discuss who was involved in the panel mentioned in line 106.
   a. Expanded upon this in lines 103-105
8. I'm concerned about the length of the survey which is 30 pages long, and wonder if the providers would have enough time from their clinical responsibilities to be able to adequately complete the survey.
   a. Discussed in lines 178-181.
9. May be creating a timeline for collecting the surveys might help with the timely analysis of the data.
   a. Added in line 256
10. It is unclear how the survey will be distributed to potential participants. Will it be sent to all movement disorder specialists, or a specific subset? How will participants be recruited?
    a. Expanded upon in lines 252-256
11. The survey development process seems to rely heavily on the input of a small group of experts. It may be beneficial to incorporate the perspectives of a broader range of clinicians and patients, as they may have different insights on barriers to guideline implementation.
    a. Excellent point, we did discuss this. Added in lines 300-302
12. It is unclear whether the survey will include any open-ended questions or opportunities for participants to provide qualitative feedback. Including these types of questions may provide more in-depth insights into the barriers and challenges faced by clinicians
    a. Added an open-ended optional question at the end of the survey as an opportunity for participants to provide feedback.

Thank you for your review of this manuscript.

Sincerely,
Jaclyn M. Martindale
